# Case report of hemochromatosis with *HJV* variation in a Chinese boy: “Non-parallel” phenomenon in phlebotomy treatment and new thinking on curative effect evaluation

**DOI:** 10.1097/MD.0000000000042626

**Published:** 2025-06-27

**Authors:** Yuhan Liu, Songyun Zhang, Xiantao Liu, Lixia Zhou, Zhuning Wang

**Affiliations:** a Department of Endocrinology & Rare Diseases, The Second Hospital of Hebei Medical University, Shijiazhuang, Hebei Province, China; b Department of Hematology, Handan First Hospital, Handan, Hebei Province, China; c Department of Radiology, The Second Hospital of Hebei Medical University, Shijiazhuang, Hebei Province, China; d Department of Endocrinology, The Second Hospital of Hebei Medical University, Shijiazhuang, Hebei Province, China.

**Keywords:** iron metabolism, juvenile haemochromatosis, liver damage, liver R2*, phlebotomy treatment

## Abstract

**Rationale:**

Juvenile haemochromatosis type 2A (JH 2A) is an autosomal recessive genetic disorder characterized by disrupted iron metabolism regulation and progressive iron overload due to *HJV* gene variation. The rapid onset and swift progression of JH 2A significantly reduce patients’ survival time. Due to the atypical clinical manifestations, early diagnosis and treatment of JH 2A pose challenges for clinical doctors.

**Patient concerns:**

An 11-year-old male, student, was hospitalized for a 2-week history of obvious fatigue accompanied by chest tightness after the activity. The patient’s chest tightness symptoms improved after rest and there was no joint pain.

**Diagnosis:**

The patient’s serum iron parameters were elevated and the liver magnetic resonance imaging (MRI) suggested liver iron overload. After genetic testing, the patient was diagnosed as *HJV* Y46X/C321X compound heterozygous mutation. He was diagnosed with JH 2A.

**Interventions:**

The patient was treated with prompt and regular phlebotomy treatment and limit the intake of foods with high iron content. The patient’s condition improved.

**Outcomes:**

The patient’s liver injury was reversed, the patient’s growth and development proceeded without further complications.

**Lessons:**

Early phlebotomy treatment can reverse the progression of the disease. Nevertheless, the nonparallel phenomenon of serum ferritin and liver iron deposition observed during maintenance treatment has prompted a reconsideration of the assessment of the curative effect of phlebotomy treatment. Liver R2* may be equally important.

## 1. Introduction

Hereditary haemochromatosis (HH) is a disease caused by the mutation of genes related to iron metabolism, which leads to excessive absorption of iron in intestinal tract, increase progressive iron load and tissue iron deposition in the body, and lead to clinical manifestations of liver fibrosis and cirrhosis, cardiomyopathy, diabetes, hypogonadotropic hypogonadism, arthropathy, skin pigmentation, etc.^[1]^ HH can be divided into 4 types,^[1]^ type 1 (HFE), type 2 (*HJV*[2A], HAMP[2B]), type 3 (TfR2) and type 4 (SLC40A1), according to the type of genetic variation. Among them, type 2 HH is also known as juvenile haemochromatosis (JH, OMIM 602390).Worldwide, cases of non-HFE haemochromatosis are rare, varying significantly in terms of ethnicity, genetics and geography. Currently, studies have mainly focused on Caucasians. Correlation studies of Asians are scarce, thus making diagnosis a challenge. JH is the most serious type of HH, and the onset age can be earlier (10–30 years old). The high incidence of cardiomyopathy and hypogonadism is its typical clinical manifestation, and heart failure is the most common cause of death.^[2]^ Most patients have a long-term unclear medical history, such as fatigue, anorexia, and amenorrhea.^[3]^ Early phlebotomy treatment has a significant curative effect and can significantly improve the prognosis.

In this retrospective study, we present the case of a patient diagnosed with JH2A.

## 2. Case report

The patient was an 11-year-old male. He was hospitalized for a 2-week history of obvious fatigue accompanied by chest tightness after the activity. He was 1.5 m tall (−1 SD to +1 SD), with a weight of 46.65 (−1 SD to +1 SD), leading the BMI of 20.73 kg/m^2^. He was of moderate nutrition. His skin and mucosa were dark in color, the secondary sexual characteristics of him were not developed. The cardiorespiratory, abdominal and nervous system examinations were normal.

Laboratory tests: Liver enzymes increased, and serum aspartate aminotransferase was 63 U/L (9.0–50.0 U/L), alanine aminotransferase was 78.8 U/L (15–40 U/L). Serum iron parameters increased, serum iron was 64.5 μmol/L (8.9–32.2 μmol/L), transferrin saturation (TSAT) was 99% (20–55%) and serum ferritin (SF) was 537.7 ng/mL (23.9–336.2 ng/mL).Thyroid, parathyroid and pancreatic islet function were normal. His sex hormones for prepubertal level, suggesting that gonadal function is not started.

Other auxiliary examinations: magnetic resonance imaging (MRI) of upper abdomen: T1WI and T2WI showed that the liver signal was significantly reduced, lower than that of the paravertebral muscle at the same level, showing a “black liver sign.” The spleen signal was normal. No abnormality was found in MRI of heart and pituitary. The results of targeted exon capture sequencing and Sanger sequencing verification showed that the *HJV* gene: c.138C>A (p.Y46X) (paternal origin), c.18G>C (p.Q6H) (maternal origin) and c.962_963delGCinsAA (p.C321X) (maternal origin). Among them, c.18G>C (p.Q6H) site is poorly conserved, and the harmfulness caused by mutation is small, so it is presumed to be a nonpathogenic mutation.^[4]^ Two other mutations are both known pathogenic mutations, but the combination of the 2 has not been reported. According to the American College of Medical Genetics and Genomics guidelines,^[5]^ it was determined to be a new pathogenic compound heterozygous variant.

**Figure 1.**
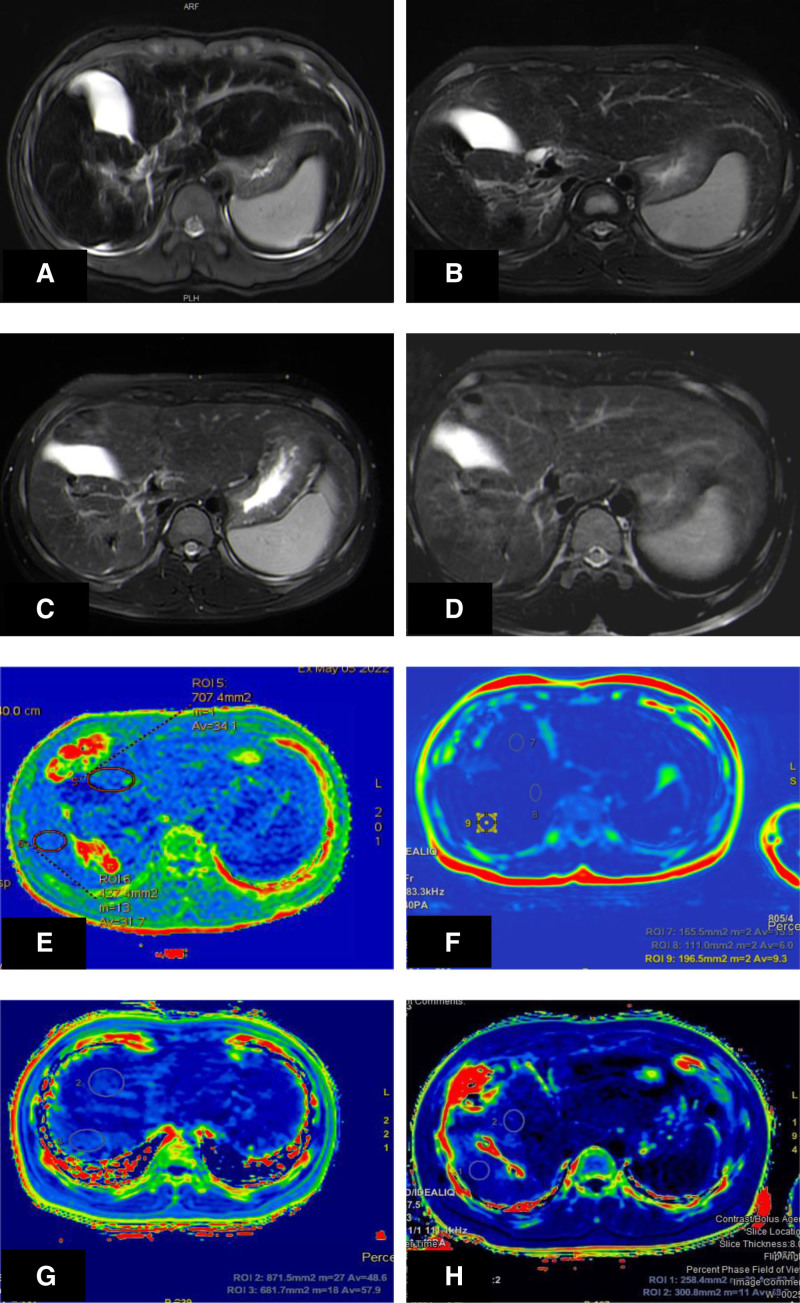
Short-axis plane of liver by T2-weighted turbo-spin echo sequence (the more the iron content, the lower the signal of the liver, and the lower the value of T2). (A) Liver MRI at initial diagnosis. (B) Liver MRI at 24 weeks of treatment. (C) Liver MRI at 48 weeks of treatment. (D) Liver MRI at 106 weeks of treatment. The results showed that the liver iron deposition was gradually improved. (E) Liver MRI measurements of R2* at 106 weeks of treatment. (F) Liver MRI measurements of R2* at 142 weeks of treatment. (G) Liver MRI measurements of R2* at 162 weeks of treatment. (H) Liver MRI measurements of R2* at 178 weeks of treatment. MRI = magnetic resonance imaging.

The patient was diagnosed with JH type 2A. The causes were as follows: The elevated serum iron parameters (TSAT > 50% and SF > 300 ng/mL) suggested iron overload; liver MRI suggests liver iron overload (Fig. 1A); the gene test results confirmed that the *HJV* gene had pathogenic variation. Further evaluation revealed that the liver fibrosis 4 index was 0.54 (<1.3 may exclude a high probability of progressive liver fibrosis^[6]^).

After diagnosed with JH type 2A, phlebotomy treatment was performed. During the induction period, phlebotomy was performed once a week, a total of 8 times. Afterwards, due to the influence of coronavirus disease 2019 epidemic, the interval of phlebotomy was slightly irregular, about once every 3 to 8 weeks. After the phlebotomy was performed for a total of 6 times, the serum iron dropped to 50 ng/mL. Then the treatment entered the maintenance period. For the next 3 years, 2 to 5 phlebotomy treatments were performed every year, and the amount of blood released each time ranged from 420 to 540 mL.

**Figure 2.**
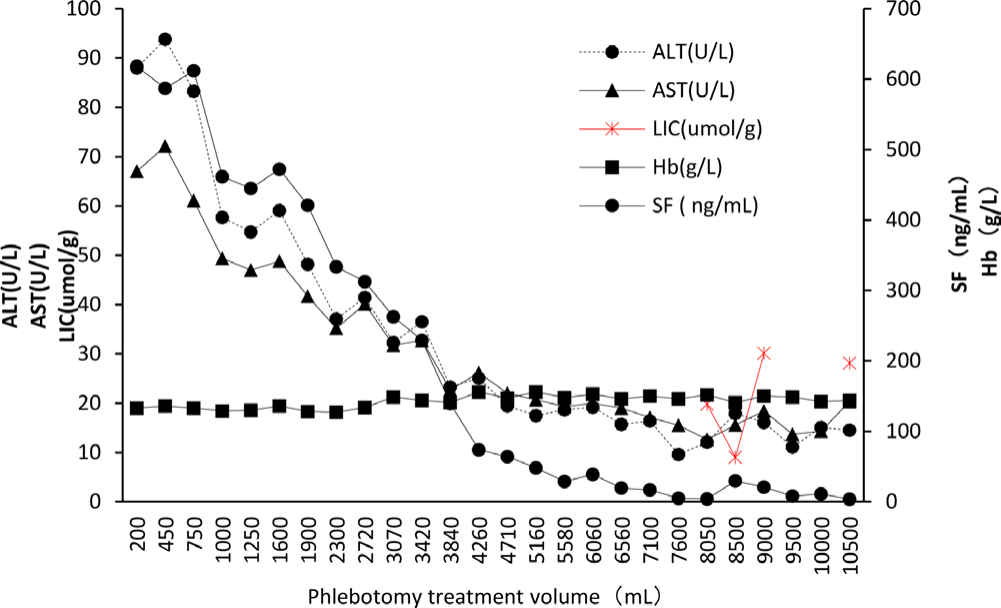
ALT/AST/LIC (left ordinate axis) and SF/Hb (right ordinate axis) of patient during phlebotomies treatment. ALT = alanine aminotransferase, AST = aspartate aminotransferase, Hb = hemoglobin, LIC = liver iron concentration, SF = serum ferritin.

Through regular treatment, remarkable curative effect has been achieved. After 5 weeks of phlebotomy treatment (the sixth week of treatment), liver enzyme indexes were back to normal range (aspartate aminotransferase 39.8 U/L, alanine aminotransferase 48.2 U/L), and the general fatigue had vanished. Additionally, his height increased dramatically, the levels of luteotropichormone and testosterone rose, the secondary sexual characteristics manifested, suggesting that puberty development proceeded as scheduled. After the 20th phlebotomy treatment (the 106th week of treatment), partial iron depletion was achieved, with serum iron decreasing to 4.3 ng/mL, TSAT normalizing to 23.67%, and liver fibrosis 4 index reducing to 0.36. Furthermore, the MRI showed no liver iron overload (Fig. 1E) (19.84 μmol/g is equivalent to 1.10 mg/g) according to the calibration formula reported by Henninger et al.^[7]^ During the subsequent phlebotomy treatment, SF fluctuated between 3.8 and 29.8 ng/mL, and liver iron concentration (LIC) fluctuated between 0.5 and 1.67 mg/g (9.02 μmol/g and 30.13 μmol/g) (Fig. 2).The latest liver hardness test showed that the liver quality was uneven, and the right lobe of the liver had a slightly strong echo in S6 segment, which was considered as uneven iron deposition, and the degree of liver fibrosis was F0 to F2 (some of them showed moderate liver fibrosis). No iron deposit was found in MRI of heart and pituitary.

## 3. Discussion

HH is an inherited disorder that disrupts iron metabolism, resulting in an accumulation of iron in different organs and severe consequences. JH is a rare but most severe form of HH, usually appearing before the age of 30.^[8]^ JH is caused by recessive mutations in either *HAMP* or *HJV*, with the *HJV* gene mutation responsible for 90% of JH cases, known as type 2A JH. The type of JH can only be diagnosed by genetic testing.^[9]^ *HJV* is a gene mapped to chromosome 1q21 that encodes hemojuvelin. It is a transcriptional regulator of hepcidin which plays a key role in regulating iron metabolism. The c.138C>A (p.Y46X) mutation (paternal origin) results in the protein only retaining the signal peptide region, but all other functional domains were lost, thus losing the protein function. The c.962_963delGCinsAA (p.C321X) mutation (maternal origin) leads to the abnormal form of HJV protein,^[10]^ which prevents the activation of hepcidin and causes abnormal iron metabolism.

One month after the symptoms appeared, the patient was diagnosed in time and treated with regular phlebotomy, and the curative effect was remarkable. Performance for the following points: Successful iron removal: TSAT and SF dropped to normal levels; MRI of the liver showed a significant reduction in iron deposition (Fig. 1B–D). Reversing liver injury: Liver enzymes returned to normal. No damage to heart and pituitary function was caused. The patient’s adolescent growth and development proceed normally. Nevertheless, for those with JH, reversing endocrine dysfunction is a more difficult task than improving liver function. Complete recovery from dysfunction is difficult with phlebotomy treatment and/or iron removal medication.^[11]^ Fortunately, our patient was diagnosed at the age of 11. Appropriate treatment was administered to him before the anterior pituitary gonadotrophin cells were impaired by iron deposition, thus precluding the emergence of hypogonadotropic hypogonadism. Normal initiation of puberty occurred, his height and secondary sexual characteristics were well developed. Meanwhile, the occurrence of other endocrine and metabolic diseases such as diabetes, thyroid diseases were avoided.

Compared with other types of HH, patients with JH have faster iron deposition in parenchyma cells and higher incidence of complications. Early diagnosis and prompt exsanguination are essential for patients with JH.^[12]^ An observational study by Prabhu et al revealed that HH patients who had phlebotomy treatment before cirrhosis or diabetes developed had a similar survival rate to that of non-affected individuals.^[13]^ For HH patients, phlebotomy treatment may reverse hepatic fibrosis and even early cirrhosis. Furthermore, patients who achieved iron depletion within 18 months of phlebotomy had significantly higher survival. The liver injury occurred in our patient when the diagnosis was made, but timely treatment quickly realized iron depletion, reversed liver injury and prevented the occurrence of serious complications such as cardiomyopathy and cirrhosis, which confirmed the importance of early diagnosis and active treatment for HH patients. Therefore, we suggest that JH should be considered for juvenile patients with unexplained liver injury.

Interestingly, our study observed a nonparallel phenomenon between serum SF and liver iron deposition during phlebotomy treatment. During the maintenance period, patients consistently exhibit SF levels below the guideline-recommended 50 ng/mL, even when experiencing iron deficiency below the lower limit of normal. However, his LIC values continue to rise gradually (Fig. 1E–H), albeit not reaching the upper limit of normal LIC value at 1.8 mg/g. Liver hardness tests also indicate moderate liver fibrosis in certain segments due to iron deposition.

This phenomenon raises the question of whether the assessment of the curative effect of intravenous phlebotomy should consider not only SF levels but also the detection of liver R2*. SF, as a reactant during the acute phase, is vulnerable to infection, inflammation, and other stressors, including cardiovascular and cerebrovascular diseases, as well as traumatic conditions.^[14]^ The signal intensity of liver MRI reflects the degree of iron deposition, based on the paramagnetic effect of iron. The relaxation rate R2* is positively correlated with LIC, making it highly sensitive and specific for diagnosing liver iron overload.^[15]^ Hence, in the monitoring of HH patients, SF should be used in conjunction with liver R2* detection to enhance the assessment of the therapeutic efficacy of phlebotomy treatment and the extent of organ damage. Additional research is necessary to ascertain if sustainable finance can be sustained amidst the continuous growth of low-income countries.

## 4. Conclusions

To sum up, we reported a case of HJVc.138C>A (p.Y46X)/c.962_963 delGCinsAA (p.C321X) with a new mutation combination. Following active intravenous phlebotomy treatment, the patient experienced resolution of symptoms, normalization of all examination indices, reversal of liver injury, and normal progression of growth and development, achieving notable results that underscore the significance of early diagnosis and treatment for JH patients. In addition, this study puts forward a new evaluation standard of curative effect in the follow-up treatment of HH patients. Given the nonparallel phenomenon between SF and LIC, the combination of liver R2* with SF could provide a more comprehensive assessment of iron overload in the organs of HH patients. Nevertheless, this conclusion warrants further validation through multi-center and large-sample clinical research.

## Author contributions

**Data curation:** Yuhan Liu, Songyun Zhang, Zhuning Wang.

**Formal analysis:** Yuhan Liu, Songyun Zhang.

**Investigation:** Yuhan Liu.

**Project administration:** Yuhan Liu.

**Resources:** Lixia Zhou.

**Writing – original draft:** Yuhan Liu, Xiantao Liu.

**Writing – review & editing:** Yuhan Liu.
